# Orthodontic Treatment Needs of 12-year-old School-going Children of Mysuru District, Karnataka, India: A Cross-sectional Study

**DOI:** 10.5005/jp-journals-10005-1531

**Published:** 2018-08-01

**Authors:** Bhagyalakshmi Avinash, Basapura M Shivalinga, Somanthan Balasubramanian, Suma Shekar, Byalakere R Chandrashekar, Battalli S Avinash

**Affiliations:** 1 Reader, Department of Orthodontics, JSS Dental College & Hospital Mysuru, Karnataka, India; 2 Professor, Department of Orthodontics, JSS Dental College & Hospital Mysuru, Karnataka, India; 3 Research Director, Department of Water and Health, JSS Academy of Higher Education & Research, Mysuru, Karnataka, India; 4 Reader, Department of Orthodontics, JSS Dental College & Hospital Mysuru, Karnataka, India; 5 Professor, Department of Preventive and Community Dentistry, JSS Dental College & Hospital, Mysuru, Karnataka, India; 6 Reader, Department of Periodontics, JSS Dental College & Hospital Mysuru, Karnataka, India

**Keywords:** Awareness, Dental health component, Esthetic component, Index of orthodontic treatment need, Malocclusion.

## Abstract

Health is the extent of functional or metabolic regulation of a living body. Many researchers have shown that oral health is directly related to the systemic condition of a person. The various researches done has shown that there is an increase in need for orthodontic treatment in most of the countries. Hence judicious planning of providing orthodontic services on a population basis is necessary to appraise the requirement of resources and manoeuvre for providing such a service.

**How to cite this article:** Avinash B, Shivalinga BM, Balasubramanian S, Shekar S, Chandrashekar BR, Avinash BS. Orthodontic Treatment Needs of 12-year-old School-going Children of Mysuru District, Karnataka, India: A Cross-sectional Study. Int J Clin Pediatr Dent 2018;11(4):307-316.

## INTRODUCTION

Oral health connects with other health systems of the body. Good oral health is important for overall well-being of an individual. Oral health is a critical component of health and hence, must be included in the provision of health care and the design of community-based programs.^1^

Improving the health and well-being of every individual will help in improving the public health of the country. Public health is nothing but a consolidation of interdisciplinary approaches of epidemiology, biostatis-tics, and health services.

Malocclusion is a misalignment or incorrect relation between the teeth of the two dental arches when they approach each other as the jaws close. Malocclusion is the most common oral health problem along with dental caries, gingivitis, and dental fluorosis.^2^

Malocclusion varies from country to country and also among different races. Studies have been done worldwide to know the prevalence of malocclusion. Nigeria,^3^ Malaysia,^4^ and Spain^5^ have reported the prevalence as 13, 37, and 41% respectively.

India is a vast and a developing country. Our country is striving to put an end to the many health-related disorders. A strong reason for this might be due to the insufficient execution of preventive health care facilities which definitely requires a strong base of epidemiological data. The results of the epidemiological studies on maloc-clusion not only helps in planning orthodontic treatment but also offers a rational approach for determining the etiological factors of malocclusions.^6^

There are various epidemiological studies on maloc-clusion in India. But there are few of them which have studied the variations of malocclusion between the states. Table 1 lists some of the states wherein the epidemiologi-cal data about malocclusion are available.^7^

There are various ways of recording malocclusion.^37,38^ Among the various types of occlusal indices, the index of orthodontic treatment need (IOTN; treatment priority) allows the categorization of malocclusion according to the level of treatment need.^39,40^

### Objectives of the Study

- To assess the severity of malocclusion in 12-year-old school-going children of Mysuru district
- To assess the awareness of malocclusion in 12-year-old school-going children of Mysuru district
- To test the agreement between the orthodontist and the child regarding the treatment need and to assess the different types of malocclusion present among 12-year-old school-going children of Mysuru district.

Keeping this as a background, this study has been undertaken to assess the orthodontic treatment need of 12-year-old school-going children of Mysuru district.

**Table 1:** **Table 1:** Epidemiology of malocclusion: Indian scenario

*State*		*Author*		*Malocclusion status*	
Himachal Pradesh		Chauhan et al^8^		31% severe malocclusion	
		Pruthi et al^9^		53% malocclusion	
Rajasthan		Trehan et al^10^		66.7%	
		Dhar et al^11^		36.42%	
Andhra Pradesh		Muppa et al^12^		14.3% class I	
				9.95% class II	
				5.33% class III	
				20.8% urban	
		Suma et al^13^		14.9% rural	
Kerala		Jacob and Mathew^14^		49.2%	
Tamil Nadu		Kannappan^15^		19.6%	
		Radha Krishna et al^16^		62.5%	
		Joseph John and Dhinaha^17^		25.1% definite malocclusion	
				6.2% handicapping malocclusion	
Delhi		Kharbanda et al^18^		91.6% class I	
				4.6% class II	
				3.4% class III	
Madhya Pradesh		Jalili et al^19^		14.4%	
Haryana		Gauba et al^20^		14.4% class I	
				13.5% class II	
				1.3% class III	
		Singh et al^21^		55.3%	
Punjab		Corruccini et al^22^ and Singh et al^23^		Crossbite	
Chhattisgarh		Ashok Kumar et al^24^ and Shetty et al^25^		2.9% definite malocclusion	
				25% severe malocclusion	
				1.4% handicapping malocclusion	
Uttar Pradesh		Singh et al,^26^ Katiyar et al,^27^ and Lew and Foong^28^		34.09%	
Maharashtra		Shaikh and Desai,^29^ Nainani and Sugandh^30^		77.9% class I	
				5.04% class II	
				2.5% class III	
Gujarat		Joshi and Makhija^31^		spacing	
Karnataka		Dinesh et al^32^		23% class I	
				4.5% class II	
				1.3% class III	
		Shivakumar et al^33^		3.7% severe malocclusion	
				15.7% moderate malocclusion	
				80.1% little/no malocclusion	
		Prasad and Savadi^34^		51.5-85.7%	
		Phaphe et al^35^		17.8% class I	
				30.1% class II	
				1.6% class III	
		Siddegowda and Rani^36^		32.8%	

## MATERIALS AND METHODS

A cross-sectional descriptive survey was planned among the school children of Mysuru district. A prior permission from the Deputy Director for Public Instructions was obtained. Also, prior permission was obtained from the concerned school authorities. The epidemiological survey was planned to be conducted in four taluks of Mysuru district. The sample size was determined using sample size formula for prevalence study. The prevalence rate was fixed at 40% and relative precision was 0.12. The sample size obtained was 840 subjects.

Two-stage sampling was planned out. In the first stage of sampling, four taluks were selected using simple random sampling by lottery method. Out of 840 subjects, 210 subjects were equally distributed to four taluks of Mysuru district. In the second stage of sampling, from each Taluk, schools were selected randomly to include 210 subjects by lottery method. In each school, children in the age group of 12 years were chosen using the class attendance register.

### Inclusion Criteria

- Children aged 12 years in the sampled schools.
- Children who provided both informed consent from parents and informed assent to participate in the study.

### Exclusion Criteria

- History of previous orthodontic treatment
- Rampant caries
- Any other craniofacial anomalies and syndromes

Informed consent and informed assent were given a week prior by the parents of the child and the child.

To know the perceived orthodontic treatment need, the esthetic component (AC) of the IOTN was used. The children were given 10 colored photographs of anterior teeth displaying various forms of malocclusion and were asked to pick photograph on the esthetic scale that most closely resembled their own dentition. The children were shown a face mirror initially which was later removed so that they refreshed the memory. The children were not allowed to look into the mirror while viewing the photograph.

To determine the normative orthodontic treatment need, in our study, we have used the modified version of IOTN. A single orthodontist carried out the examination. Type III examination as recommended by the American Dental Association, which includes inspection using mirror and a probe done under good illumination, was conducted. The examination was performed under natural light in the school premises using disposable gloves and mouth mirrors. A periodontal probe was used for millimeter measurement. Sufficient number of autoclaved instruments was carried to the examination site to avoid the interruption during the study.

For each of these two assessment tools, IOTN dental health component (DHC), IOTN-AC, patients were categorized into three groups as having (Table 2):

1. Little/no orthodontic treatment need
2. Moderate orthodontic treatment need
3. Definite orthodontic treatment need

At the end of the clinical examination, a school oral health education lecture was given to all the children in the school to create awareness about dental health and orthodontic treatment.

### Statistical Analysis

Data were transformed into Statistical Package for the Social Sciences (SPSS) Windows version 16, where cleaning, coding, recoding, crosschecking, and processing and analysis were done by the statistician.

The following statistical tests were applied.

- Frequency
- Descriptive
- Cross-tabulations (Contingency table analysis)
- Chi-square test
- Kappa statistic

**Table 2:** **Table 2:** Levels of treatment need

		*Little/no need*		*Moderate need*		*Definite need*	
IOTN-DHC		1-2		3		4-5	
IOTN-AC		1-3		4-6		7-10	

All the statistical methods were carried out through the SPSS for Windows (version 16.0).

## RESULTS

The results of the study will be discussed under the following headings:

### Prevalence of Malocclusion in Mysuru District

Out of the 845 participants, 409 (48.4%) were boys and 436 (51.6%) were girls.

### Orthodontic Treatment need in Relation to Gender

**Graph 1:**
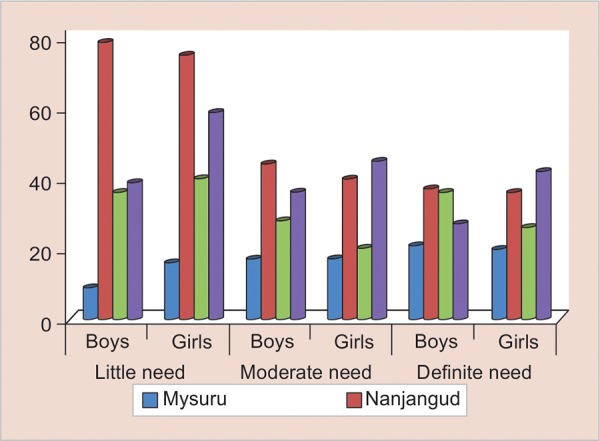
Orthodontic treatment need based on IOTN-DHC in relation to gender in four taluks of Mysuru district

The tabular and graphical representation is shown in Table 3 and Graph 1 respectively.

From Table 3 and Graph 1 it is observed that

- The participants were from rural-based taluks than the urban (Mysuru taluk).
- The maximum numbers of participants were from government schools rather than private aided and private unaided schools.
- The maximum participants were girls compared with boys.
- The malocclusion/IOTN classification reveals that among 409 boys, 163 (39.9%) had little need for orthodontic treatment, while 125 (30.6%) had moderate need, and 121 (29.6%) had definite need for orthodontic treatment. Among 436 girls, 190 (43.6%) had little need for orthodontic treatment, while 122 (28%) had moderate need, and 124 (28.4%) had definite need for orthodontic treatment.
- There was no statistically significant difference with regard to orthodontic treatment need between male and girl study participants in the present study (p = 0.53).
- This was true even when a separate comparison was made between boys and girls in Mysuru taluk (p = 0.44), Nanjanagud taluk (p = 0.98), Hunsur taluk (p = 0.35), and T-Narsipur taluk (p = 0.76).

### Study of Awareness about Orthodontic Treatment Need in Mysuru District

**Graph 2:**
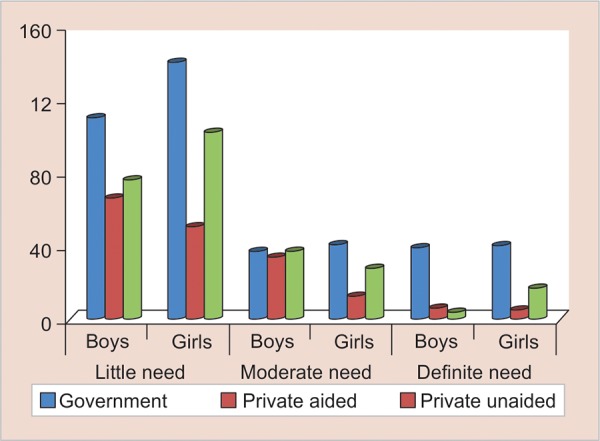
Orthodontic treatment need based on IOTN-AC in relation to gender and type of school

The EC of IOTN was used to know the perceptive level of orthodontic treatment need (Table 4 and Graph 2).

### Orthodontic Treatment Need in Relation to Type of School

Among 186 male participants from government schools, 110 (59.1%) had little need, 37 (19.9%) had moderate need, and 39 (21%) had definite need for orthodontic treatment. Among 221 girls from government schools, 140 (63.3%) had little need, 41 (18.6%) had moderate need, and 40 (18.1%) had definite need for orthodontic treatment. There was no significant difference between boys and girls with regard to ECs of IOTN in government schools (p = 0.67, Table 4 and Graph 2).

This was true even when a separate comparison between boys and girls was done in private aided (p = 0.17) school. However, definite orthodontic treatment need was significantly higher among girl participants when an exclusive gender-wise comparison was made in private unaided schools (p = 0.03).

Conclusion—When compared with the different types of schools, it is observed that private unaided school children were mainly concerned with dental appearance than the government and private aided school children. This is due to the fact that children drawn from private unaided schools are from upper economically favored society.

**Table 3:** **Table 3:** Orthodontic treatment need based on IOTN-DHC in relation to gender in four taluks of Mysuru district

		*Little need*				*Moderate need*				*Definite need*				*Total*					
*Taluk name*		*Boys, n (%)*		*Girls, n (%)*		*Boys, n (%)*		*Girls, n (%)*		*Boys, n (%)*		*Girls, n (%)*		*Boys, n (%)*		*Girls, n (%)*		*Statistical Inference*	
Mysuru		9 (36.0) (19.1)		16 (64.0) (30.2)		17 (50.0) (36.2)		17 (50.0) (32.1)		21 (51.2) (44.7)		20 (48.8) (37.7)		47 (47.0) (100)		53 (53.0) (100)		χ^2^: 1.63, df: 2, p: 0.44	
Nanjangud		79 (51.3) (49.4)		75 (48.7) (49.7)		44 (52.4) (27.5)		40 (47.6) (26.5)		37 (50.7) (23.1)		36 (49.3) (23.8)		160 (51.4) (100)		151 (48.6) (100)		χ^2^: 0.048, df: 2, p: 0.98	
Hunsur		36 (47.4) (36.0)		40 (52.6) (46.5)		28 (58.3) (28.0)		20 (41.7) (23.3)		36 (58.1) (36.0)		26 (41.9) (30.2)		100 (53.8) (100)		86 (46.2) (100)		χ^2^: 2.12, df: 2, p: 0.35	
T-Narsipura		39 (39.8) (38.2)		59 (60.2) (40.4)		36 (44.4) (35.3)		45 (55.6) (30.8)		27 (39.1) (26.5)		42 (60.9) (28.8)		102 (41.1) (100)		146 (58.9) (100)		χ^2^: 0.553, df: 2, p: 0.76	
Total		163 (46.2) (39.9)		190 (53.8) (43.6)		125 (50.6) (30.6)		122 (49.4) (28.0)		121 (49.4) (29.6)		124 (50.6) (28.4)		409 (48.4) (100)		436 (51.6) (100)		χ^2^: 1.28, df: 2, p: 0.53	

**Table 4:** **Table 4:** Orthodontic treatment need based on IOTN-EC

		*Little need*				*Moderate need*				*Definite need*				*Total*					
*Type of school*		*Boys, n (%)*		*Girls, n (%)*		*Boys, n (%)*		*Girls, n (%)*		*Boys, n (%)*		*Girls, n (%)*		*Boys, n (%)*		*Girls, n (%)*		*Statistical Inference*	
Government		110 (44.0) (59.1)		140 (56.0) (63.3)		37 (47.4) (19.9)		41 (52.6) (18.6)		39 (49.4) (21.0)		40 (50.6) (18.1)		186 (45.7) (100)		221 (54.3) (100)		χ^2^: 0.81 df: 2 p: 0.67	
Private aided		66 (56.9) (62.3)		50 (43.1) (73.5)		34 (72.3) (32.1)		13 (27.7) (19.1)		6 (54.5) (5.7)		5 (45.5) (7.4)		106 (60.9) (100)		68 (39.1) (100)		χ^2^: 3.56 df: 2 p: 0.17	
Private unaided		76 (42.7) (65.0)		102 (57.3) (69.4)		37 (56.9) (31.6)		28 (43.1) (19.0)		4 (19.0) (3.4)		17 (81.0) (11.6)		117 (44.3) (100)		147 (55.7) (100)		χ^2^: 9.81 df: 2 p: 0.01	
Total		252 (46.3) (61.6)		292 (53.7) (67.0)		108 (56.8) (26.4)		82 (43.2) (18.8)		49 (44.1) (12.0)		62 (55.9) (14.2)		409 (48.4) (100)		436 (51.6) (100)		χ^2^: 7.17 df: 2 p: 0.03	

**Table 5:** **Table 5:** Association between normative and perceptive orthodontic treatment need among study participants

		*Perceptive need*							
*Normative need*		*Little need, n (%)*		*Moderate need, n (%)*		*Definite need, n (%)*		*Total*	
Little need		301 (85.3) (55.3)		43 (12.2) (22.6)		9 (2.5) (8.1)		353 (100) (41.8)	
Moderate need		130 (52.6) (23.9)		99 (40.1)		18 (7.3) (16.2)		247 (100) (29.2)	
Definite need		113 (46.1) (20.8)		48 (19.6) (25.3)		84 (34.3) (75.7)		245 (100) (29.0)	
Total		544 (64.4) (100)		190 (22.5) (100)		111 (34.3) (100)		845 (100) (100)	
*Nominal by nominal—*		*Measure of agreement—*		*Assmp.Std Error^a^*		*Approx.* T^b^		*p-value*	
*Cramer’s V*		*Kappa*							
0.355		0.319		0.025		13.853		<0.001	

As the prevalence rate of malocclusion at normative level and perceptive level is understood, it is necessary to know how well the child’s and orthodontist’s opinions match with respect to the treatment need and also to know which type of malocclusion is most frequent in Mysuru district.

### Agreement between the Orthodontist and the Child and the Frequency and Types of Malocclusion in Mysuru District

There was a weak but statistically significant agreement between normative orthodontic treatment need which is assessed by DHCs of IOTN with the perceptive orthodontic need which was assessed by ECs of IOTN (Kappa coefficient—0.319, p < 0.001) (Table 5).

### Frequency of Different Types of Malocclusion

Demographics play an important role in assessing the type of malocclusion. For example, Caucasians are known to have class III tendency and people of African origin tend to have bimaxillary protrusion. In India, even though there is a mix of different ethnic groups, the population in Kerala has tendency toward bimaxillary protrusion.

**Graph 3:**
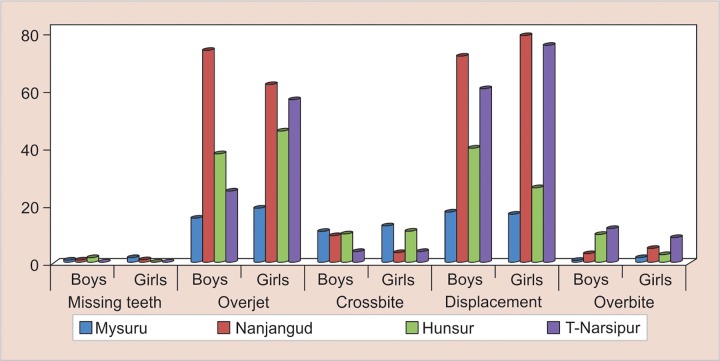
Distribution of various malocclusion traits in relation to gender among participants in four taluks of Mysuru district

Among 409 boys, 4 (1%) had missing teeth, 153 (37.4%) had an increased overjet, 35 (8.6%) had crossbite, 191 (46.7%) had displacement, and 26 (6.4%) had overbite. Among 436 girls, 3 (0.7%) had missing teeth, 184 (42.2%) had an increased overjet, 32 (7.3%) had crossbite, 198 (50.9%) had displacement, and 19 (4.4%) had overbite. Displacement of teeth was the most common malocclu-sion trait followed by an increase in overjet among both boys and girls in the present study with missing teeth being the least prevalent. However, the difference in the distribution of these malocclusion traits between boys and girls was not statistically significant (p = 0.48). This was evident even when a separate comparison was made among participants from Mysuru (p = 0.94), Nanjangud (p = 0.38), Hunsur (p = 0.07), and T-Narsipur taluk (p = 0.07) (Table 6 and Graph 3).

## DISCUSSION

Children of 12 years age have a good capacity to remember, retrieve, and apply information related to specific events and experience. Psychological theories believe that bullying victims are often socially isolated and suffer from psychological problems including anxiety and depression.^41^ Name calling and teasing in childhood can leave lasting impressions extending into adulthood. People, who are satisfied with their facial appearance and presumably their dental appearance, seem to be more self-confident and have higher self-esteem than those who are dissatisfied with their facial appearance.^42^ Severe malocclusions were associated with feelings of uselessness, shamefulness and inferiority.^37^

### Prevalence of Malocclusion in Mysuru District

The present study was designed to provide information about the orthodontic treatment needs and the prevalence of malocclusion among 12-year-old school-going children.

In the present study, 58.2% of the subjects were in need of orthodontic treatment. A similar study in Travancore population^43^ reported 53.3% in need of orthodontic treatment. However, studies done by Singh et al,^44^ Amado et al^45^ showed 68.4 and 83.8% in need of orthodontic therapy. This difference could be because the latter studies were done on the higher age-range subjects. Also, since the latter studies are done on subjects in Himachal Pradesh and Kerala, there might be racial differences.

On the contrary, studies done on Japanese popula-tion,^46^ Iranian population,^47^, and American population^48^ showed the even higher percentage of malocclusion. The greater percentages of malocclusion from these studies may be due to racial variations, different sample size, genetic predisposition, differences in lifestyle, and variation in growth and facial skeleton.

In the present study, boys had a higher need for orthodontic treatment (60.2%) as compared with girls (53.6%). But this was not statistically significant (p = 0.53, Table 3 and Graph 1). Similar results were obtained in the studies done by Otuyemi et al,^3^ Onyeaso and Sanu,^49^ and Onyeaso.^50^ However, this contrasts the finding of Esa et al^4^ on Malaysian children, and Naveen Kumar et al^51^ on Davangere children. This difference could be due to variation in the dentofacial morphology for boys and girls globally. Also, there were no statistically significant differences in relation to gender in each taluk.

**Table 6:** **Table 6:** Prevalence of various malocclusion traits in relation to gender among participants in four taluks of Mysuru district

		*Missing teeth*				*Overjet*				*Crossbite*				*Displacement*				*Overbite*				*Total*					
*Taluk name*		*Boys, n (%)*		*Girls, n (%)*		*Boys, n (%)*		*Girls, n (%)*		*Boys, n (%)*		*Girls, n (%)*		*Boys, n (%)*		*Girls, n (%)*		*Boys, n (%)*		*Girls, n (%)*		*Boys, n (%)*		*Girls, n (%)*		*Statistical inference*	
Mysuru		1 (33.3) (2.1)		2 (66.7) (3.8)		16 (45.7) (34.0)		19 (54.3) (35.8)		11 (45.8) (23.4)		13 (54.2) (24.5)		18 (51.4) (38.3)		17 (48.6) (32.1)		1 (33.3) (2.1)		2 (66.7) (3.8)		47 (47.0) (100)		53 (53.0) (100)		X^2^: 0.76, df: 4, p: 0.94	
Nanjangud		1 (50.0) (0.6)		1 (50.0) (0.7)		74 (54.4) (46.2)		62 (45.6) (41.1)		10 (71.4) (6.2)		4 (28.6) (2.6)		72 (47.7) (45.0)		79 (52.3) (52.3)		3 (37.5) (1.9)		5 (62.5) (3.3)		160 (51.4) (100)		151 (48.6) (100)		X^2^: 4.20, df: 4, p: 0.38	
Hunsur		2 (100) (2.0)		0 (0) (0)		38 (45.2) (38.0)		46 (54.8) (53.5)		10 (47.6) (10.0)		11 (52.4) (12.8)		40 (60.6) (40.0)		26 (39.4) (30.2)		10 (76.9) (10.0)		3 (23.1) (3.5)		100 (53.8) (100)		86 (46.2) (100)		X^2^: 8.54, df: 4, p: 0.07	
T-Narsipura		0 (0) (0)		0 (0) (0)		25 (30.5) (24.5)		57 (69.5) (39.0)		4 (50.0) (3.9)		4 (50.0) (2.7)		61 (44.5) (59.8)		76 (55.5) (52.1)		12 (57.1) (11.8)		9 (42.9) (6.2)		102 (41.1) (100)		146 (58.9)		X^2^: 6.97, df: 4, p: 0.07	
Total		4 (57.1) (1.0)		3 (42.9) (0.7)		153 (45.4) (37.4)		184 (54.6) (42.2)		35 (52.2) (8.6)		32 (47.8) (7.3)		191 (49.1) (46.7)		198 (50.9) (45.4)		26 (57.8) (6.4)		19 (42.2) (4.4)		409 (48.4) (100)		436 (51.6) (100)		X^2^: 3.49, df: 4, p: 0.48	

### Awareness about Orthodontic Treatment need in Mysuru district

The second objective of this study was to know the perceptive orthodontic treatment need. To assess this, the EC of index of orthodontic treatment need (AC-IOTN) was used. Only 35.6% of the subjects felt that they are in need of orthodontic treatment. This means that the subjects prefer to get orthodontic treatment not because of functional concerns or to prevent the loss of tissues within the oral cavity but because of the consequences of the esthetic impairment caused by malocclusion. The inconsistencies highlighted between the child’s perception and normative needs in our study are supported by the findings of de Oliveira and Sheiham^52^ and de Oliveira et al.^53^ The explanation for this could be that some children have remarkable levels of concerns for minor discrepancy, whereas others are more tolerant of major occlusal problems. The finding of this study is in accordance with the studies done by Al-Sarheed et al,^54^ Kerosuo et al,^55^ Kok et al,^41^ and Ghijselings et al,^56^ but contrary to the study done by Onyeaso and Arowojolu,^57^ whose study found a higher percentage of perceptive need among their subjects, i.e., 81.7%. This difference might be because of the inclusion of wide age range of the subjects (12-18 years).

The study found that girls were more aware regarding orthodontic treatment and this was statistically significant (Table 4 and Graph 2). This is in accordance with the study done by Jung.^58^

### Agreement between the Orthodontist and the Child and the Frequency and the Types of Malocclusion in Mysuru District

Several studies have indicated that patients overestimate their pretreatment condition more than the clinicians. When the agreement between the orthodontist and the child was checked, there was a weak agreement (kappa coefficient: 0.319, p < 0.001, Table 6) between the orthodontist and the child. This means that the child’s self-perception and the orthodontist’s perception of the subjects categorized most of the subjects as having mild treatment need (child-determined IOTN-AC, 64.4% mild; orthodontist-determined IOTN-DHC, 41.8% mild). Our results are in agreement with those of Abu Alhaija et al,^59^ who found that students between 13 and 17 years of age were more inclined to rate themselves as having no need of treatment. Kolawole et al^60^ also found that a higher percentage of children perceived their malocclusions on the attractive end of the esthetic scale (92%) while the orthodontist found 37.6% in moderate-to-definite treatment need. This clearly shows that the awareness about the presence of malocclusion is less.

When the measurement of agreement between the orthodontist and the child with respect to gender was checked, there was a statistically significant difference in girls (kappa coefficient: 0.344, p < 0.001, Table 5). The measurement of the agreement was higher in girls compared with boys. Although malocclusion is dependent on gender, when it comes to the understanding between the orthodontist and the child about the disorder, it was evident that girls agreed with the professional judgment compared with boys. This difference might be because girls tend to be more cooperative and understanding. According to Amoda et al,^45^ Cucalon and Smith,^61^ girls exhibit a significantly greater level of cooperation with orthodontic treatment. Southard et al^62^ also found that girls were more cooperative when the Millon Adolescent Personality Inventory was applied to the orthodontic patients.

### Frequency of Different Types of Malocclusion

In the present study, 58.2% children presented with malocclusion. Distribution of malocclusion in population showed that a maximum number of children, i.e., 46%, presented with displacement, 39.9% presented with increased overjet, 7.9% presented with crossbite, 5.3% presented with increased overbite, and 0.8% presented with missing teeth. The increased frequency of displacement and overjet in the study population can be explained by this being ascribed to the reduction in the jaw size with evolution and due to the transition of diet from coarse to soft. These results are in accordance with the results of other studies by other researchers.^63^ Our finding that displacement is the most common feature (46%) contrasts the with that of Siddiqui et al^64^ who found increased overjet to be the most common trait leading to malocclusion. A contributing factor for this difference is that Siddiqui et al^64^ studied orthodontic patients presenting to a clinic. Increased overjet is an obvious sign of malocclusion in one’s mouth and patients presenting to clinics will be to some extent aware of their malocclusion status, whereas patients presenting displacement may or may not be aware of their clinical malocclusion. The results of our study are also supported by a study done by Borzabadi-Farahani et al.^65^

There was no statistically significant difference between the type of malocclusion and gender. This is in accordance with the study done by Grand et al,^66^ Nadim et al,^67^ Reddy et al,^68^ Onyeaso,^69^ Kaur et al,^70^ and Lauc.^71^

## CONCLUSION

The observations recorded from our study are as follows:

### Severity of Malocclusion

- Little/no need for orthodontic treatment which may or may not require orthodontic treatment is observed in 41.8% of the subjects with 43.7% in girls and 39.9% in boys.
- Moderate need for orthodontic treatment which requires treatment is observed in 29.2% of the subjects with 27.8% in girls and 30.6% in boys.
- Definite need for orthodontic treatment where treatment is mandatory is observed in 29% of the subjects with 28.5% in girls and 29.6% in boys.

### Awareness of Orthodontic treatment

- The awareness about orthodontic treatment need is in 35.6% of the subjects with 32.9% in girls and 38.4% in boys.
- The awareness of little/no need for orthodontic treatment is in 64.4% of the subjects with 67.1% in girls and 61.6% in boys.
- The awareness of moderate need for orthodontic treatment is observed in 22.5% of the subjects with 18.6% in girls and 26.4% in boys.
- The awareness of definite need is observed in 13.1% of the subjects with 14.3% in girls and 12% in boys.
- There is a lack of agreement between the orthodontist and the child which indicates that the awareness about the malocclusion and its treatment is lacking in the society.

### Frequency of Malocclusion

The malocclusion parameters as recorded by the modified version of IOTN is as follows:

- Missing teeth is observed in 0.8% of the subjects.
- Overjet is observed in 39.9% of the subjects.
- Crossbite is observed in 7.9% of the subjects.
- Displacement is observed in 46% of the subjects.
- Overbite is observed in 5.3% of the subjects.

### Limitations of the Study

- Children other than 12 years of age were not included.
- Awareness of the parent toward orthodontic treatment is not included.
- It is only an observational study and no intervention is done.
